# Supplementary material for: Evaluation of the Association of Recombinant Proteins NanH and PknG from Corynebacterium pseudotuberculosis Using Different Adjuvants as a Recombinant Vaccine in Mice
Source: Vaccines (Basel). 2023 Feb 23;11(3):519. doi: 10.3390/vaccines11030519 (PMC10051533; doi:10.3390/vaccines11030519)
Supplement: Supplementary file 1 [file vaccines-11-00519-s001.zip › vaccines-2210461-supplementary.pdf]

## Supplementary Material

**ID manuscript vaccines-2210461: "Evaluation of the association of recombinant proteins NanH and PknG from *Corynebacterium pseudotuberculosis* using different adjuvants as a recombinant vaccine in mice"**

**Nicole Ramos Scholl [1], Mara Thais de Oliveira Silva [1], Tallyson Nogueira Barbosa[1], Rodrigo Barros de Pinho [1], Mirna Samara Dié Alves [1], Ricardo Wagner Portela [2], Vasco Ariston de Carvalho Azevedo [3], Sibele Borsuk[1*]**

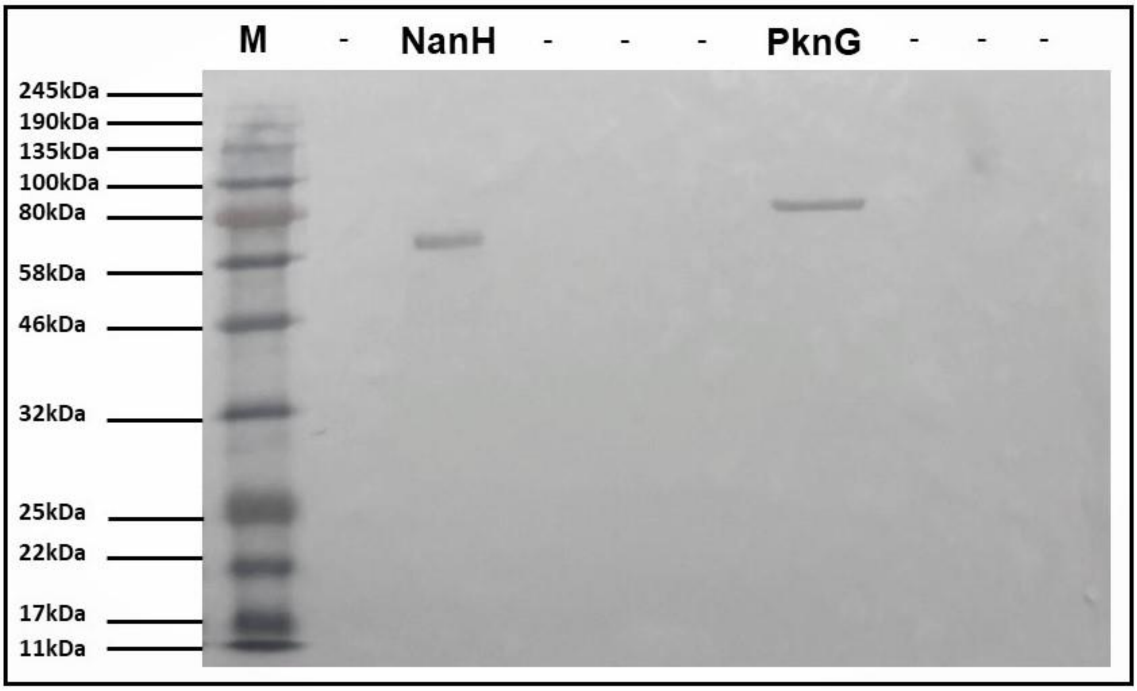


**Figure S1.** Identity characterization of the recombinant proteins NanH and PknG. Western Blot analysis of NanH and PknG proteins using an anti-6x His tag monoclonal antibody (Sigma Al-drich). (1) Pre-stained protein ladder; (2) purified rNanH; (3) purified rPknG and (-) are blanks. rNanH and rPknG are shown as reactive bands of approximately 70 kDa (2) and 80 kDa (3), respectively.
